# Hardware-Efficient Configurable Ring-Oscillator-Based Physical Unclonable Function/True Random Number Generator Module for Secure Key Management

**DOI:** 10.3390/s24175674

**Published:** 2024-08-31

**Authors:** Santiago Sánchez-Solano, Luis F. Rojas-Muñoz, Macarena C. Martínez-Rodríguez, Piedad Brox

**Affiliations:** Instituto de Microelectrónica de Sevilla, IMSE-CNM, CSIC/Universidad de Sevilla, 41092 Sevilla, Spain; macarena@imse-cnm.csic.es (M.C.M.-R.); brox@imse-cnm.csic.es (P.B.)

**Keywords:** hardware security, physical unclonable functions, true random number generators, programmable devices, intellectual property modules, secure key management

## Abstract

The use of physical unclonable functions (PUFs) linked to the manufacturing process of the electronic devices supporting applications that exchange critical data over the Internet has made these elements essential to guarantee the authenticity of said devices, as well as the confidentiality and integrity of the information they process or transmit. This paper describes the development of a configurable PUF/TRNG module based on ring oscillators (ROs) that takes full advantage of the structure of modern programmable devices offered by Xilinx 7 Series families. The proposed architecture improves the hardware efficiency with two main objectives. On the one hand, we perform an exhaustive statistical characterization of the results derived from the exploitation of RO configurability. On the other hand, we undertake the development of a new version of the module that requires a smaller amount of resources while considerably increasing the number of output bits compared to other proposals previously reported in the literature. The design as a highly parameterized intellectual property (IP) module connectable through a standard interface to a soft- or hard-core general-purpose processor greatly facilitates its integration into embedded solutions while accelerating the validation and characterization of this element on the same electronic device that implements it. The studies carried out reveal adequate values of reliability, uniqueness, and unpredictability when the module acts as a PUF, as well as acceptable levels of randomness and entropy when it acts as a true random number generator (TRNG). They also illustrate the ability to obfuscate and recover identifiers or cryptographic keys of up to 4096 bits using an implementation of the PUF/TRNG module that requires only an array of 4×4 configurable logic blocks (CLBs) to accommodate the RO bank.

## 1. Introduction

The exponential growth in the number of electronic devices that send and receive information through the Internet to access a wide variety of services focused on electronic commerce and administration, health, or leisure, to name just some of the most important sectors, has led to the emergence of new types of crime, the so-called “cyber-crimes”, related to the access or modification of private information, the denial of services in critical facilities, or the impersonation of users or systems [1,2,3].

The vulnerability of Internet of Things (IoT) devices to different types of cyber attacks constitutes a major drawback to the Internet, since it very negatively affects the activities of citizens, organizations, and governments [4,5,6]. For this reason, increasing the security of systems to ensure the confidentiality, integrity, and availability of data, as well as the authenticity of devices that process and transmit them, has become one of the main design challenges, especially in the case of devices with limited resource capacity and whose manufacturing costs do not make it feasible to include expensive security elements for key storage or the implementation of complex cryptographic algorithms [7,8].

To face some of the mentioned threats, physical unclonable functions (PUFs) of different natures and based on different operating principles have been used in recent years to generate identifiers and keys linked to the devices in which they are implemented, as well as to store the latter without need of using high-cost nonvolatile memories. In silicon PUFs, their operation is founded on the variability intrinsic to the manufacturing process of integrated circuits (ICs) and how it affects differently in each of the chips the delay paths of a given circuit structure (in the case of delay-based PUFs, such as ring oscillator (RO) [9], arbiter [10], or butterfly [11] PUFs), or the initial value of a memory element (in memory-based PUFs, such as SRAM [12] or DRAM [13,14] PUFs).

From a security point of view, the three main characteristics that determine the performance (or quality) of a PUF are: unpredictability, uniqueness, and reliability [15,16,17,18,19]. Unpredictability is related to the difficulty in predicting, even for its designers and manufacturers, the behavior of the PUF once it is implemented on a chip. It can be estimated based on other related properties, such as uniformity, randomness, or entropy. Uniqueness establishes the extent to which two different instances of the PUF provide two different outputs when the same inputs and configuration parameters are applied to them. Finally, reliability measures the ability of a PUF to return the same outputs, or at least outputs close enough that they can be correctly recovered using an error correction code (ECC), when successive invocations are made to the PUF.

This last characteristic ensures the recovery of the secret information generated by the PUF as many times as needed, while the first two determine the strength of a PUF against possible attacks. The security of strong PUFs (those that can provide a large number of challenge–response sequences) can be threatened by machine-learning-based attacks. To combat this threat, numerous solutions have been proposed in recent years to improve the nonlinearity of the PUF structure or to obfuscate the challenge–response sequence, both for Arbiter-PUFs [20,21,22] and RO-based PUFs [23,24]. On the other hand, prediction-based attacks are not applicable to weak PUFs (with a limited number of challenge–response sequences), although they can be vulnerable to different types of side-channel attacks based on power consumption or electromagnetic emissions [25].

Unpredictability, uniqueness, reliability, and attack-resistance make the PUF a key element in building a hardware root of trust (RoT) linked to the silicon device. This RoT can be used as an anchor for successive levels of a trust chain that should reach the application software accessible to users of the system or service [26].

However, as an electronic circuit, the performance of a PUF is typically determined by two other parameters. As in most electronic systems, one of these parameters is related to the response speed, that is, the time that elapses from the moment the PUF operation is invoked until the output is produced. To make comparisons as fair as possible, this parameter should be normalized based on the number of output bits of the PUF. The other important parameter is what is commonly called ‘hardware efficiency’, defined as the relationship between the number of bits provided in the PUF output and the silicon resources required for its implementation. For RO-PUFs, hardware efficiency is typically measured by the ratio between number of ROs and the resources used to implement them.

RO-based PUFs are the ones that are most widely reported in the literature [27,28,29,30,31,32,33,34,35,36,37,38,39,40,41,42,43,44,45,46,47,48,49,50,51,52,53,54,55,56,57,58,59], especially that related to field-programmable gate arrays (FPGAs), not only because of their special suitability to be implemented on these devices but also because they generally present acceptably good uniqueness and reliability values. However, its main drawback lies in the need to use a large amount of resources to achieve in its output the high number of bits required by the current cryptographic algorithms. In recent years, there have been numerous proposals that take advantage of different types of configurability to increase the number of ROs in a PUF. This increase makes it possible to use some selection mechanism to choose the most appropriate ROs in order to improve the reliability of the PUF while allowing the hardware efficiency of the circuit to be augmented by providing a greater number of bits in its output.

This paper describes the development of a security primitive, with dual functionality as a PUF and a true random number generator (TRNG), that takes full advantage of the structure of the current programmable devices offered by the Xilinx Series 7 families [60] (AMD, Santa Clara, CA, USA) to leverage the configurability of the basic building blocks in the FPGA fabric to multiply by 128 the hardware efficiency of the RO block used in previous proposals from the authors [27,28]. In this work, the increase in hardware efficiency is used to meet two complementary objectives. First, to carry out the characterization of the properties of the proposed PUF/TRNG module on a significantly high number of samples to ensure the statistical significance of the results. Second, to undertake a new practical design of the PUF/TRNG that requires a smaller amount of resources while considerably increasing the number of output bits compared to other previous proposals.

To achieve these objectives, we have designed two intellectual property (IP) modules that incorporate the configurable RO block as the core of both realizations, and are implemented, in the Xilinx Zynq-7000 System-on-Chip (SoC) device available on the Pynq-Z2 development board [61] (TUL, Taipei, Taiwan), 13 test systems with different configuration parameters that incorporate several instances of each of these IPs connected through an AXI4-Lite bus to the ARM dual core processing system available on the device. Furthermore, the software library introduced in [28] has been adapted to the new designs and completed with new functions and applications that facilitate the online realization of the different tasks of characterization and performance estimation for both modules. These facilities also illustrate the use, in a key management system, of a version of the proposed PUF/TRNG that includes a configurable RO block that occupies only 16 configurable logic blocks (CLBs) of the device and is capable of generating and correctly recovering cryptographic keys up to 4096 bits in length.

In summary, this work presents hardware and software components that provide a complete practical solution, efficient both in terms of performance and resource consumption, for the incorporation of two basic functionalities to increase the security of embedded systems implemented in programmable devices. Among its main contributions, it is worth highlighting the following:

- The proposal of an optimized RO block structure that takes full advantage of the CLBs of current Xilinx FPGA and SoC families to significantly increase the hardware efficiency of previous designs.
- The encapsulation of the design as a highly parameterizable IP module, with dual PUF/TRNG functionality, connectable through a standard interface that facilitates its integration with soft- or hard-core general-purpose processors for the implementation of embedded systems.
- The development of a library of low- and high-level software components that facilitate the interaction of the processor with the hardware and make it possible to carry out online the successive stages of validation, characterization, and exploitation of the design.
- The implementation of 13 test systems with different implementation parameters and the collection of a considerable amount of experimental data using different run-time options to perform a complete characterization of the statistical properties of the adopted solution, as well as to obtain the metrics to evaluate the performance of the proposed PUF/TRNG module.
- The availability of an open access repository that includes hardware and software components necessary so that the reader can perform different experiments on their own development board to analyze the effect of different design parameters on the capacity of the PUF/TRNG module to act as an essential element for the obfuscation and recovery of secret information in a key management system.

After introducing the motivations and objectives of the work and advancing its main contributions, the remainder of the document is structured as follows. Section 2 first introduces the operating principle and general structure of RO-based PUFs. Next, an extensive review of the proposals in the literature that apply the concept of configurability to somehow improve the performance of RO-PUFs is carried out, indicating the similarities and discrepancies between the different solutions and establishing a taxonomy that allows them to be classified into various groups. Our proposal to include a configurable RO block in the PUF/TRNG module is introduced in Section 3, where the main aspects of the operation of the two IP modules developed in the work are also detailed. The incorporation of these IPs into two groups of test systems implemented on a Pynq-Z2 development board, as well as the description of the main results derived from the set of tests carried out on each of them, constitutes the contents of Section 4 and Section 5, respectively. As a demonstration of the usefulness of our proposal, Section 6 illustrates the use of the proposed PUF/TRNG module as a basic element of a RoT supporting a key management system. Finally, the main conclusions derived from carrying out the work are summarized in Section 7.

## 2. Configurable RO-PUFs

Initially introduced in [9], a RO-PUF bases its operation on the differences in the oscillation frequencies of a set of ring oscillators with the same number of inverter stages and with identical connection schemes (i.e., the same layout). A priori, the frequency of all ROs should be the same. However, as a consequence of the variability introduced in the different stages of the IC manufacturing process, once the chip is manufactured, each RO has a unique, unpredictable, and different characteristic frequency from that of the other ROs that form the PUF and from that of the equivalent ROs on chips located in different positions on the same silicon wafer or in the same position on other wafers from the batch.

**Figure 1:**
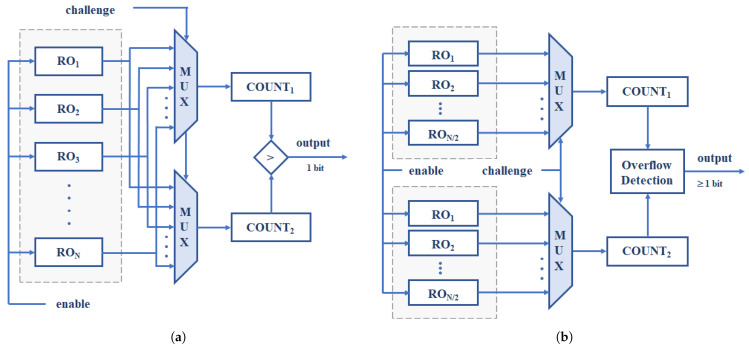
Conventional block diagrams of RO-PUFs whose outputs consist of (**a**) one bit for each pair of ROs compared [9] and (**b**) more than one bit per comparison [30].

In practice, a real PUF such as the one illustrated in Figure 1a includes a sufficiently high number of ROs, grouped in a single block or distributed in a pair of them, and operates based on a challenge–response mechanism. The input of the PUF (challenge sequence) is a set of bit strings that determine which ROs are to be compared with each other and in what order, while the output (response) consists of another bit string formed by the concatenation of the sign bits resulting from comparing the pairs of ROs indicated by the challenge sequence.

The size of the PUF therefore depends on the number of comparisons between ROs established by the challenge sequence and the number of bits obtained from each comparison (N/2 and 1, respectively, in [9], *N* being the number of ROs). To increase the length of the PUF response, the number of comparisons can be increased to N−1 without compromising the correlation between the output bits [29]. The PUF output length for a given RO bank size can also be increased if more than one bit is obtained in each comparison [30,31], as is the case in the design whose block diagram is illustrated in Figure 1b. And even more so if two comparison strategies are combined, as occurs in the proposals described in [27,28]. It is worth highlighting that the latter also allows the response speed of the PUF to be doubled.

The incidence of sources of electronic noise of different natures, the possible changes in the operating conditions of the circuit, and the probable deviations from its original behavior due to aging force the PUFs to be subjected to different enrollment processes throughout its useful life cycle. To improve the behavior of the PUFs in terms of reliability, in these processes, it is usually common to include some type of selection mechanism that allows choosing those comparisons (or ROs) that provide the best values of the metrics used to evaluate the performance of the module. In this sense, the availability of a large number of ROs is also key to discarding those that are less favorable without significantly limiting the length of the PUF output.

The exploitation of the concept of configurability, understood as the possibility of selecting between different design variants that do not significantly affect the structure of a circuit but do give rise to changes in its behavior, has been widely used in the literature to improve the performance of PUFs, both in terms of its security features (mainly reliability) and the efficiency of its hardware implementation (the number of output bits and/or the amount of challenge–response sequences).

**Figure 2:**
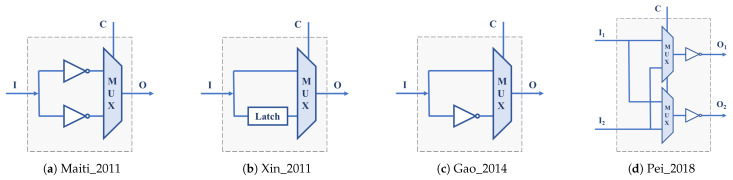
Configurable delay elements used in different RO-PUFs and implemented from the basic components of the CLBs: (**a**) Maiti et al. (2011) [33]. (**b**) Xin et al. (2011) [34]. (**c**) Gao et al. (2014) [35]. (**d**) Pei et al. (2018) [36].

Many of these proposals take advantage of the logical resources available in the basic building blocks of programmable devices offered by different manufacturers to select between alternative delay paths that cause different oscillation frequencies in the ROs and, consequently, different behaviors in the PUF. The RO-PUF described in [32,33] leverages the resources of a Xilinx Spartan-3E FPGA to implement a 3-stage RO using a single CLB. The inverter used in each stage is selected between two possibilities using a configuration bit (Figure 2a), so that each RO supports eight different configurations, which allows for selecting the frequencies of the RO pairs to be compared according to the 1-out-of-8 scheme proposed in [9] without the need to increase by eight the resources required to implement the RO bank. In order to take even more advantage of the FPGA resources, ref. [34] uses the flip-flops available in the CLBs of a programmable device of the same family to increase the number of configuration bits to 8 (Figure 2b), so that the resulting PUF can provide 256 different identifiers with the same resources as the PUF in [9]. Reorganizing the data provided by the authors of the previous works, ref. [35] analyzes a scheme of 16 15-stage ROs in which each inverter, once characterized in frequency, can be included or not in the RO with the idea of improving PUF reliability (Figure 2c). The structure provides as many configuration bits as stages make up the ring, although it must be taken into account that only configurations with an odd number of inverter stages are valid.

The FPGAs and SoCs supplied by Xilinx include among the components of their CLBs n—input memories used as look-up tables (LUTs) capable of implementing any logic function with *n* inputs or two independent logic functions sharing n−1 inputs. The value of *n* and the number of LUTs per CLB depend on the family of programmable devices. Ref. [36] takes advantage of the two outputs of the 6-input LUTs available in the Xilinx Spartan-6 family FPGAs to design an ‘OR construction’ unit that allows for selecting the inverters of the two 7-stage ROs that can be implemented in a single CLB of these FPGAs (Figure 2d), thus doubling the hardware efficiency of the previous proposal.

**Figure 3:**
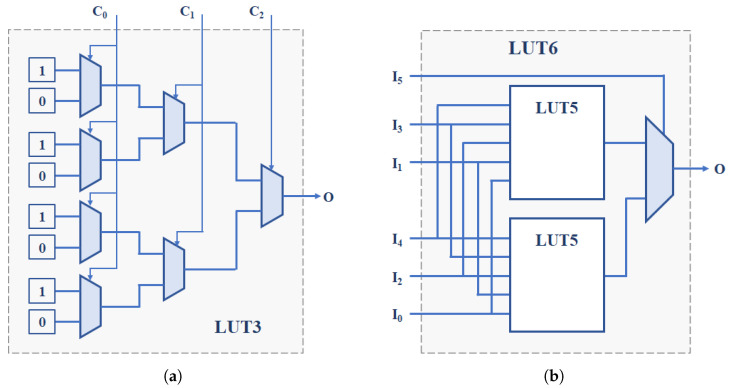
Programmable Delay Line: (**a**) Programmable delay inverter introduced in [62]. (**b**) 6-input LUT of Xilinx 5, 6, and 7 Series programmable devices.

The straightforward use of LUTs to implement programmable delay lines (PDL) applied to the PUF design was initially introduced in the Arbiter-PUF described in [62] (Figure 3a) and then exploited in recent years to build other types of PUFs, including many configurable RO-PUFs. In [37], it is used to implement ROs with three configuration bits on a Spartan-3E FPGA. In combination with a thresholding technique to eliminate comparisons that can produce ‘bit flips’, 283 bits are obtained from a PUF that incorporates 130 of these ROs. The study described in [38] takes advantage of the 6-input LUTs of the Xilinx 5, 6, and 7 Series families (Figure 3b) to extract 31 bits of entropy from two ROs by calculating second-order differences that provide good uniqueness and reliability values to the resulting PUF. In [39], five LUT inputs connected to each other (course PDL) or a single input with the other four connected to 0 (fine PDL) are used as the configuration bit of each of the RO stages implemented on a Xilinx Spartan 6 device. The sequence of challenges, generated in this case using a Galois linear feedback shift register (LFSR), is used to select both the ROs to compare and their configuration. The ROs are distributed in two blocks so that it is possible to compare all the ROs in one block with all the ROs in the other block using different configurations, avoiding correlations.

The six inputs of the LUTs of the Xilinx Virtex-6 family are used in [40] to meet the double objective of improving 16× the hardware efficiency of the PUF and increasing the frequency differences in the ROs to be compared in order to improve reliability (reaching 100% in a wide range of voltages and temperatures according to the authors). Ref. [41] describes the use of a combined technique of first-order differences and difference in sums of differences that allow for doubling the hardware efficiency of the proposal in [38], requiring half the resources to obtain 32 bits of two ROs implemented on a Xilinx Kintex-7 device. The PUF proposed in [42] uses a modern FPGA from the Kintex-7 family to implement a structure similar to that used in [39], but now, five of the six inputs of the LUTs are used as independent control inputs in the PDL coarse. To obtain each output bit, a temporal majority voting (TMV) technique is applied by calling the PUF 15 times with the same configuration and calculating the XOR of all the possible configurations. The same number of configuration bits per LUT is used in the CRO described in [43]. Combined with a 1-out-of-n frequency selection scheme similar to those in [9,33], this proposal achieves reliability values very close to 100% at the cost of worsening the uniqueness of the PUF. Finally, as an additional example of work that applies the PLD concept to the PUF implementation, ref. [44] analyzes the influence of using different inputs on the LUTs of devices from the Xilinx Zynq-7000 SoC family.

Describing the behavior of the delay elements of a configurable RO by linking it to the concept of PDL implemented through LUTs or defining it by a set of logical components that provide identical functionality implemented on the same LUTs is only a matter of interpretation. However, while the first approach is almost exclusively associated with the world of programmable devices, the second is also applicable to any of the application-specific integrated circuit (ASIC) manufacturing technologies. Multiplexers, such as those shown in the delay elements of Figure 2, are basic components that are usually present in the CLBs of the different families of FPGAs or whose functionality can be easily implemented in the LUTs of these devices. However, their implementation in ASICs is not very efficient, so different alternatives have been proposed to build and design PUFs whose implementation is efficient in both FPGAs and ASICs. Many of them are based on a cascade structure of 2-input XOR gates, so that one of these inputs determines whether the value of the other input or its negation is propagated to the output.

**Figure 4:**
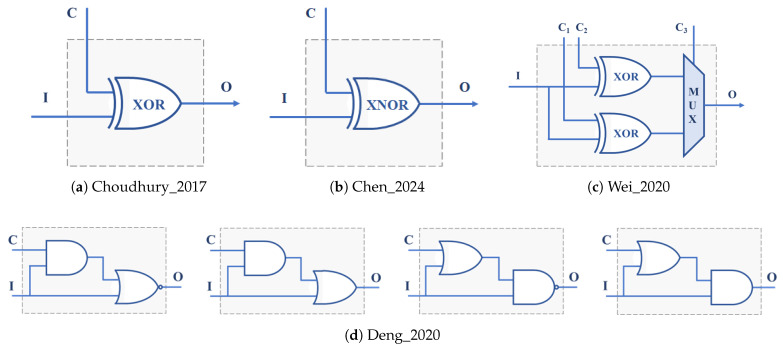

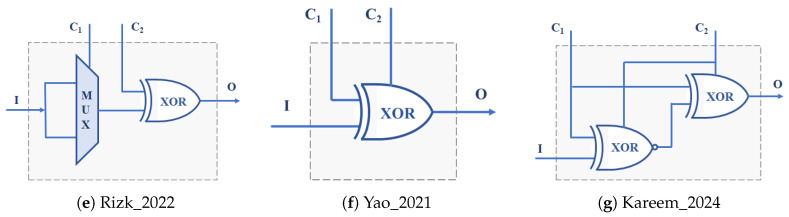
Configurable delay elements using logic gates for FPGA and ASIC implementation: (**a**) Choudhury et al. (2017) [45], Zhang et al. (2017) [46], and Liu et al. (2019) [47]. (**b**) Chen et al. (2024) [48]. (**c**) Wei et al. (2020) [49]. (**d**) Deng et al. (2020) [51]. (**e**) Rizk et al. (2022) [50]. (**f**) Yao et al. (2021) [53]. (**g**) Kareem et al. (2024) [54].

A PUF that uses a configurable RO built with four XOR gates and a functional block, which makes the total number of inversions in the loop odd for the ring to oscillate, is analyzed in [45] (Figure 4a). The RO has four configuration bits, and its implementation occupies a CLB of a Xilinx Artix-7 FPGA. The configurable RO used in [46,47] also uses a CLB, in this case, of a Spartan-6 device, to include seven XOR gates and one AND gate. The seven configuration bits provide 64 valid configurations (those with an odd number of ones), considerably increasing the hardware efficiency of the PUF. A similar structure implemented in Xilinx Zynq-7000 devices, incorporating *n* XNOR gates that can be configured as inverters or buffers to select the ring oscillation frequency (Figure 4b), plus an XOR gate to guarantee that the total number of inverter stages is odd, is used in [48] to build a PUF with good reliability and uniqueness values.

The transformer PUF described in [49] uses three of the six multiplexers available in each CLB of the Artix-7 family to increase the number of configuration bits of an RO composed of an enable stage, formed by an AND gate and a XOR gate, and three stages consisting of two XOR gates and a multiplexer (Figure 4c). Depending on the input to the XORs, the structure can behave as a configurable RO-PUF (CRO-PUF) or as a bistable ring PUF (BR-PUF). The number of configuration bits of this CRO is 10; however, there are only 64 combinations of the configuration bits that make the circuit oscillate, so its hardware efficiency is similar to that of [46,47]. The proposal in [50] uses 5 LUTs and 4 MUXes from the Xilinx Spartan 7 family of CLBs to implement a CRO with 8 configuration bits (Figure 4e). With the idea of avoiding the use of 2-input multiplexers, whose implementation in an IC requires four logic gates, in [51], different delay stages composed of two gates are proposed to implement configurable RO-PUFs in ASICs or FPGAs (Figure 4d). The PUF was implemented in TSMC 38 nm technology, as well as in FPGAs of the Artix-7 family. The configurable RO-PUF in [52] uses these delay blocks together with a MUX that allows the number of CRO stages to be selected and a mechanism to enhance reliability and reduce the sensitivity of the circuit to environmental conditions.

Some hybrid solutions that can be included in this category have also been proposed, such as the one that appears in [53], which combines the ideas of XOR gates and PDLs (Figure 4f) to design a PUF with 8-stage ROs that admit 214 configurations and can be implemented in a single CLB of the Xilinx Virtex-6 family. To increase the number of CRO configurations, the PDL concept is also applied to the delay stage formed by a pair of XNOR and XOR gates (Figure 4g), proposed in [54]. In addition to increasing hardware efficiency, the data presented by the authors show that this solution outperforms many of the existing related works.

**Figure 5:**
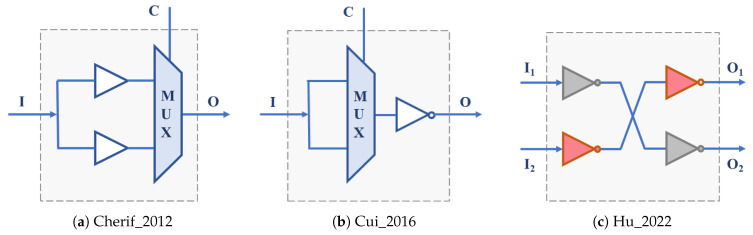
Self-comparison RO-PUFs: (**a**) Cherif et al. (2012) [55]. (**b**) Cui et al. (2016) [56]. (**c**) Hu et al. (2022) [58].

In all the PUFs discussed so far, two counters are used to compare the oscillation frequencies of pairs of ROs that are active simultaneously. An alternative option, based on the fact that the use of different configurations of a CRO gives rise to oscillations of different frequencies, consists of sequentially comparing the frequencies associated with two successive configurations of a single CRO. The Loop-PUF described in [55] bases its operation on the use of *N* delay chains fed back by an inverter to form a configurable RO implemented on an Cyclone II FPGA (Intel, Santa Clara, CA, USA). Each chain is made up of *M* stages in each, of which one of the two possible delays is selected by means of a configuration bit (Figure 5a), so that the structure can oscillate with 2×M×N different frequencies (although from this number, it is necessary to eliminate the configurations that give rise to equivalent challenges). The configurable RO proposed in [56] combines delay units consisting of a two-input multiplexer and a single inverter (Figure 5b). Implemented on a Spartan-6 FPGA, the selection inputs of the multiplexers of *N* successive stages provide 2×N configurations whose oscillation frequencies can be compared two by two following a self-comparison strategy. The same self-comparison strategy is used in the CRO based on the idea of PDL associated with LUTs described in [57], which also uses adjustable count time to maximize the stability of PUF responses. Finally, with the idea of mitigating the presence of bias in the PUF output caused by systematic variations in the IC manufacturing process, ref. [58] uses an interleaved structure that uses two LUTs of an Artix-7 device to implement four inverters with eight configuration bits in each stage and employs a comparison scheme based on two passes and two phases (Figure 5c).

## 3. Hardware-Efficient Configurable RO-PUF/TRNG Module

The RO-PUFs described in [27,28] use a CLB of Xilinx Series 7 devices to implement four ROs, each composed of three inverter stages and an enable stage. The simultaneous application of two RO selection strategies, obtaining two bits for each pair of compared ROs, and the possibility of choosing different options related to the distance between the ROs involved in each comparison, the size of counters, and the bits selected to make up the output, allows the construction of a very versatile element with double functionality PUF/TRNG and acceptable hardware efficiency. However, without giving up the other features, the hardware efficiency of this module can be considerably increased if the capacity of the basic components of the programmable devices is fully exploited to implement two configurable ROs in each of the CLBs of the RO bank, according to the structure described below.

### 3.1. Structure and Main Components of the PUF/TRNG

**Figure 6:**
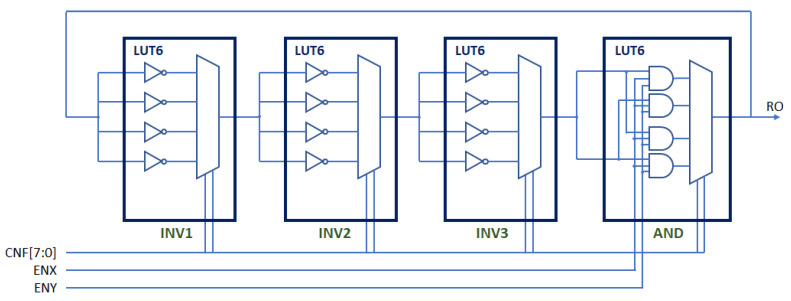
Configurable RO with two enable signals and eight configuration bits implemented on half of the LUTs available in CLBs of Xilinx 7 Series programmable devices.

Figure 6 shows the schematic of a configurable RO formed by three inverter stages and an enable stage. Each of the inverter stages implements four inverters that share the same input and whose output is selected through the two control bits of a multiplexer. The last stage of the structure, which has two inputs to facilitate the row and column enablement of the elements of a RO bank, allows the closure of the RO feedback loop through four alternative paths selectable by two control bits. The entire structure can be replicated twice using the eight 6-input LUTs available in each CLB of Xilinx Series 7 devices. It is worth highlighting that the functionality of the multiplexers that appear in the figure is implemented in the LUT itself, without implying the need for additional logical or connection resources of the programmable device.

The structure therefore provides a total of eight configuration bits that allow 256 configurations to be selected, which is equivalent to being able to implement 512 different ROs in each CLB of the Xilinx Series 7 devices. This structure increases 128 times the number of ROs per CLB with respect to that described in [28] (Configuration entries should be the same in all CLBs to ensure that ROs with identical layout are compared).

The design of the pair of CROs is carried out with AMD Vivado Design Suit using a structural VHDL description that includes LUT6 primitives, as well as a series of directives to allow the existence of combinational loops, without which Vivado would not perform the synthesis process, and to set the connectivity of the different elements so that all the CROs of the PUF have the same layout, and therefore, the differences in the operating frequencies depend exclusively on the variations in the manufacturing process and the existence of some kind of electronic noise. The same procedure is also used to implement a bank of configurable ROs (cro_bank), located in a compact matrix of CLBs, whose size and position in the programmable logic of the device can be defined by the designer using parameters when synthesizing and implementing the circuit. The VHDL code that describes this block also includes placement directives to make the location of successive CROs within the CRO bank follow a snake pattern, thus ensuring that the relative distances between successive pairs of ROs defined by the sequence of challenge are always the same.

Unlike most RO-PUFs proposed in the literature, which use an external reference clock to compare the frequencies of a pair of ROs and provide a single bit to the output in each comparison, in the PUF/TRNG module described in this work, two comparisons between ROs are carried out in parallel, each of which provides two bits at the output of the PUF, thus achieving a factor of 4× in hardware efficiency compared to more conventional structures. Performing two simultaneous comparisons exploits the two different behaviors identified in [59], depending on whether the two compared ROs are implemented in LUTs located in the same position of different CLBs or whether both ROs are placed in LUTs located in different positions within the same or a different CLB. Verifying that this double behavior is maintained when using the configurable RO proposed in this work as a basic component of the RO bank is one of the objectives of the experimental tests described in Section 4.

**Figure 7:**
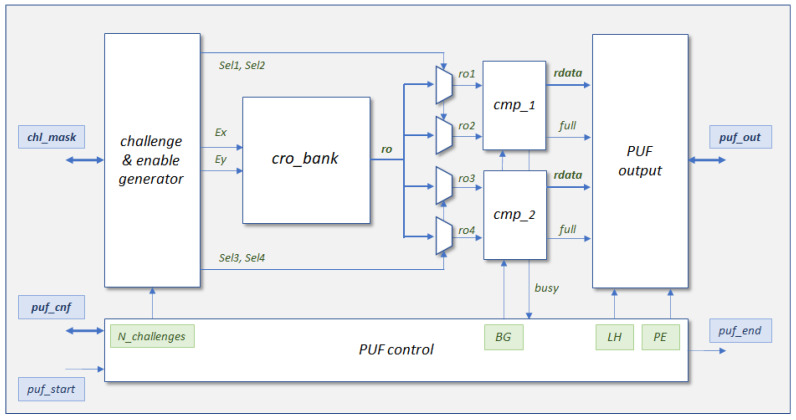
Block diagram of the proposed configurable RO-PUF/TRNG core (blue boxes represent IO signals and buses; green boxes show selectable run-time options).

The main components of the PUF/TRNG are shown in the simplified block diagram in Figure 7. Once the start signal of the operation (puf_start) is received by the core, the control block that orchestrates the entire operation of the module manages the block that generates the sequence of challenges (Sel1,⋯Sel4) and enable signals (Ex,Ey) to select and activate, respectively, the two pairs of RO involved in each comparison cycle. When generating the challenge sequence, this block can discard the comparisons that most negatively affect the reliability of the module when acting as a PUF. This challenge selection mechanism is based on a selection mask obtained in an enrollment process prior to using the device.

Using as input the signals from the four ROs selected by the multiplexer bank (ro1,⋯ro4), the two comparisons are carried out in parallel, using two comparison blocks (cmp_1,cmp_2) that incorporate two counters that are incremented until one of them reaches the maximum possible value according to their size (which is another of the design parameters set by the designer before synthesizing and implementing the circuit). To prevent the end-of-count signals from being affected by delay differences in binary counters, the comparison blocks always use Gray code counters, constructed from the corresponding binary counters, to generate these signals. However, the bits that make up the output of the system, whether acting as PUF or TRNG, can be taken from both types of counter depending on the value assigned to the BG (Binary/Gray) run-time option.

Two other run-time options, defined by the user when invoking the module, allow the selection of its functionality as PUF or TRNG (PE, PUF/Entropy source) and choosing whether uniqueness or reliability is prioritized in the first of the cases (LH, Lower/Higher). The setting of these options determines which part of the information resulting from the two comparisons will be included in the response provided by the output block. This block is also responsible for concatenating the partial responses of the successive comparison cycles and storing the output in a series of 32- or 64-bit registers (another parameter defined by the designer) to facilitate its reading by the SoC processor. The amount of data that makes up the output, and therefore, the number of registers required, also depends on which of the two possible alternative modes is defined when synthesizing and implementing the design. In ‘characterization mode’, the logic values of the signals indicating which counter has reached the maximum capacity (full) and the entire contents of the slowest counter (rdata) in each of the two comparisons are included in the output. As we see in Section 4, this mode is used in the early stages of the development process to analyze the behavior of the different bits to determine which of them to use when implementing the module in ‘operation mode’, in which only the four selected bits are added to the output of the PUF/TRNG in each comparison cycle.

The comparison cycle continues while either of the two comparators is completing their function and repeats until the number of challenges (N_challenges) defined by the user when invoking the module is reached, after which the puf_end signal is generated to indicate that the module is ready to provide its output and wait for a new invocation. The rest of the blocks that make up the core of the PUF/TRNG are similar in structure and operation to those used in [28] (the reader can consult this reference for a detailed description of each of them).

### 3.2. PUF/TRNG IP Module Development Process

As shown in Figure 7, in addition to the ports corresponding to the start and end of operation, the PUF/TRNG core has three other I/O channels that allow the configuration of different run-time options, writing the challenge selection mask, and reading the response, respectively. To facilitate the integration of the PUF/TRNG into SoC solutions, these ports are accessible to the processing system through a set of memory-mapped registers using a standard interface based on the AXI4-Lite bus [63]. Once added to the Vivado IP catalog, the developed IP module can be incorporated into a design using the environment provided by the Vivado IP Integrator tool. A graphical user interface (GUI) allows the user to define the various design parameters: size and location of the CRO block, number of counter bits, width of the communication buses, and operation mode.

As mentioned previously, replacing the RO bank included in the PUF/TRNG described in [28] by a bank of configurable ROs such as the one shown in Figure 6 allows the hardware efficiency of the module to be multiplied by 128. Depending on the intended application for the device, this increase can be used to reduce the size of the module, increase the number of bits in its response, or combine both objectives. However, in any case, the first question that arises is to verify the behavior of these configurable ROs compared to those used in the previous designs.

**Figure 8:**
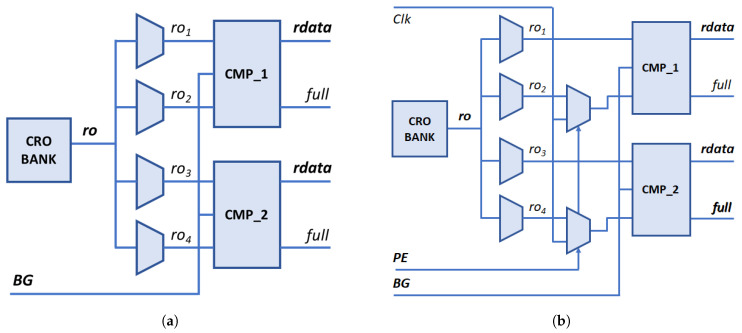
Configuration of puf4r5_1.0 in operation (**a**) and characterization mode (**b**).

A first version of the IP (referred to as puf4r5_1.0 in the text) was initially designed to achieve this objective. As mentioned above, the module can be implemented in two modes of operation: characterization and operation. In this version of the IP, the characterization mode has a double utility. On the one hand, the evaluation of the bits of the counters used in the comparisons is carried out in order to select the most appropriate ones for the functionality of the design acting as PUF or TRNG. On the other hand, and as a novelty in this version, it is possible to obtain the oscillation frequencies of the different ROs for each of their configurations. To do this, a pair of multiplexers is used (controlled by the PE parameter, which has no other function in this operation mode) that allow the clocks provided by each of the RO configurations to be compared with a reference clock (in our case the same system clock). Figure 8 shows how the assignment of the input signals to the comparison blocks is carried out in the two implementation modes. In both the operation and characterization modes, when PE=1, the inputs to the four counters of the comparison blocks come from the four ROs that intervene in each comparison cycle. In characterization mode, with PE=0, the inputs to the upper counters also correspond to ro1 and ro3, while a reference clock with a frequency lower than expected in any of the ROs (the 100 MHz system clock) is connected to the inputs of the lower counters. In this way, the values captured in the slower counters allow the frequencies of all the ROs implemented to be estimated for each possible configuration.

As demonstrated by the results that appear in the next Section, the statistical study carried out on test systems that incorporate the puf4r5_1.0 IP module showed that regardless of the RO configuration used, all the resulting PUFs present a behavior similar to that of the previous designs with regard to their reliability (i.e., the repeatability of the response), uniqueness (differences between the outputs of different PUF instances), and unpredictability (entropy and absence of bias in the PUF responses). These reasons justified the design of a new version of the IP module, puf4r5_2.0, which takes advantage of the configurability of ROs to considerably increase the hardware efficiency of the design, simultaneously achieving the objectives of reducing the programmable logic resources it consumes and increasing the number of bits it provides. The main difference between the two versions is that in the former, configuration signals must be provided by software when the IP operation is invoked, while in the latter, they are generated internally as part of the process of generation of challenges and enable signals. This implies the simplification of the design of this block.

## 4. Test System for Statistical Characterization of the CRO-Based PUF/TRNG

Given that most of the metrics that characterize the behavior of PUFs and TRNGs are statistical in nature, one of the main problems that designers of this type of circuits face is having a high number of samples that ensure significance statistics of the results obtained from the experimental data. In the case of PUFs implemented on programmable devices, this requirement is usually resolved by instantiating different copies of the design at different locations of the device and/or using several FPGA development boards. However, it is difficult to find contributions in which data collected on more than a hundred different samples appear.

**Figure 9:**
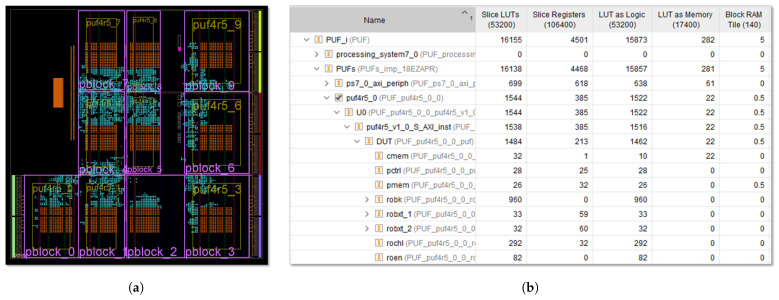
Test system for statistical characterization of the CRO-based PUF/TRNG: (**a**) Distribution of 10 instances of the puf4r5_1.0 IP on the programmable logic of the SoC device. (**b**) Resource consumption of the test system and the different components of the IP module.

The test system shown in Figure 9 takes advantage of the configurable RO structure proposed in Section 3 to greatly overcome this drawback by including 10 instances of the puf4r5_1.0 IP module, which allows the analysis of a total of 2560 PUFs or TRNGs with different behaviors using a single development board (Pynq-Z2 in this case). The location of the CRO banks of the different copies of the IP is defined when instantiating each of them, while that of the remaining elements of the PUF is freely chosen by the synthesis and implementation tool within the areas defined by the designer through ‘pblock’ directives. Each instance incorporates a bank of 8×15 CLBs, which implement 240 CROs (with 256 possible configurations per RO). Comparing the frequencies of pairs of ROs with the same configuration, it is possible to obtain up to 960 bits in the output of each instance.

The system is completed with the communications infrastructure (AXI Interconnect) required to connect the different IPs with the ARM dual-core processor included in the fabric of the Zynq-7000 device present on the Pynq-Z2 board [61]. To analyze the influence of the design parameters, the test systems have been implemented with IPs in characterization and operation mode that use 10, 12, and 14 bits in the counters involved in the comparison between the oscillation frequencies of the ROs.

The availability of a powerful application processor and the support of the Linux operating system provided by the PYNQ [64] project have been very useful for the development of different software components that facilitate not only the control of the PUF/TRNG operation but also the possibility of carrying out, on the development board itself, the exhaustive characterization of its properties and the evaluation of the metrics that define the module performance.

To carry out these tasks on the test systems described in this article, the software development kit (SDK) presented in [28] has been adapted and completed with the idea of taking into account the configurability of the ROs used and including new functionalities for behavioral analysis and statistical characterization of the proposed structures. The new version of the SDK is also structured into three levels: (1) drivers to facilitate the interaction between the software and the hardware that implement the PUF/TRNG functionality; (2) functions to configure and control the operation of the module; and (3) applications to evaluate the different metrics that determine its performance and illustrate its use in security tasks. Table 1 shows a list of the main functions and applications included in the SDK for puf4R5 IP modules.

The results summarized in the following sections have been obtained by executing a complete set of tests based on these applications on the test systems mentioned above.

### 4.1. ROs Oscillation Frequencies

As previously commented, puf4r5_1.0 includes a mechanism that allows the estimation of the oscillation frequency of the different ROs. To do this, the IP module must be implemented in characterization mode, and the puf_bitselect application is used. The idea is to apply a reference clock, whose frequency is lower than that expected in all the ROs, to one of the inputs of the comparators so that the values of the counters provided in each comparison cycle will be proportional to the frequency of two of the ROs compared in each cycle. The frequency of an RO is calculated according to Equation (1), taking into account that when its counter has reached the maximum value, C_max, the counter associated with the reference clock of 100 MHz has reached the value C_ref.

(1) $$f_{RO}=100\times \frac{C_{max}}{C_{ref}}$$

**Figure 10:**
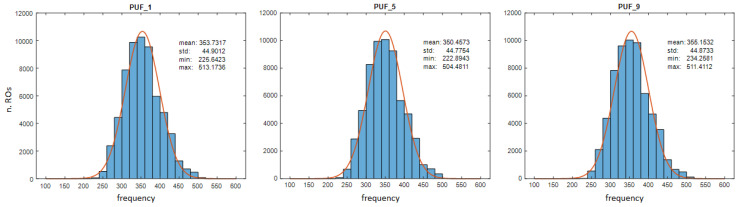
Oscillation frequencies of the ROs for 3 of the 10 instances of the puf4r5_1.0 IP module included in the test system. Each histogram includes the frequencies corresponding to the 256 configurations of the 240 CROs of the IP.

The results obtained by analyzing the behavior of all the configurations of each of the 240 CROs contained in the 10 IPs instantiated in the test system show that oscillation frequencies depend on both the location of the ROs and the configuration used. It can be seen that the oscillation frequencies present a normal distribution centered around 350 MHz and with a standard deviation of about 45 MHz. As an example, Figure 10 shows the histograms of the oscillation frequencies of the ROs for three of the IPs included in the test system. To obtain maximum precision in the measurements, the tests have been carried out in this occasion using the 15-bit counters of a test system implemented expressly for this purpose. However, as was logically expected, the values obtained by repeating the tests in test systems that use 10-, 12-, and 14-bit counters do not present significant variations.

The fact that the different configurations of the same RO use different delay paths and, in short, present different oscillation frequencies, allows us to assume that various configurations could be combined to increase the number of comparisons carried out to obtain the output of a hardware-efficient PUF/TRNG module. However, before undertaking this task, it is important to analyze in more detail the outputs of modules with different configurations and verify that all of them present characteristics similar to those reported in [28]. These tests are described in the following sections.

### 4.2. PUF/TRNG_1.0 Bit Selection

To obtain more than one bit in each RO comparison, it is necessary to use a bit selection mechanism that allows for choosing the most appropriate ones for each of the functionalities of the PUF/TRNG module [31,59]. The puf_bitselect application provided in the SDK for the puf4r5_1.0 IP facilitates the evaluation of the average values of stability, probability, and entropy of each of the bits (sign and counter associated with the lowest frequency RO) resulting from each comparison in the different IP instances included in a test system implemented in characterization mode.

**Figure 11:**
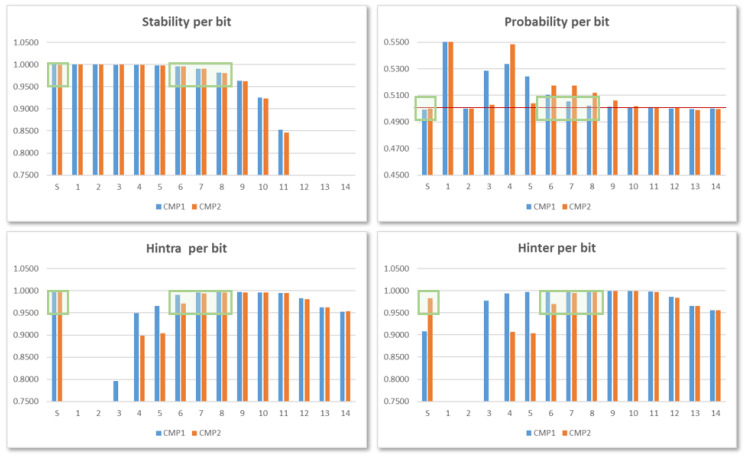
Stability, probability, and entropy metrics calculated for each bit of the counters (average values for one hundred calls to all PUF/TRNG instances and RO configuration options, with the two types of counters; green boxes point out the most suitable bits to construct the output for PUF functionality).

Figure 11 shows the results corresponding to invoking one hundred consecutive times each of the 10 IP instances in the test system that uses 14-bit counters, performing the maximum number of comparisons, and using the 256 possible configurations of the ROs to obtain data equivalent to 2560 PUF/TRNG samples with 960-bit outputs. The different metrics follow a behavior similar to that described in [28]: (1) stability values decrease in the direction from MSB to LSB, whereas the Hintra values grow in the same direction; (2) Hinter for the sign bit only reaches an acceptably high value in the second of the comparisons; and (3) probability values of the least significant bits are close to the ideal value of 0.5.

These data allow us to corroborate the conclusions obtained in the previous works to maintain the bit selection criterion used in them. As the green boxes in the graphics point out, for PUF functionality, the most suitable bits to construct the output correspond to two of the bits 6–8, for comparisons between ROs implemented in LUTs placed in different CLB locations (COMP1), and the sign bit plus one of bits 7–8 in the other case (COMP2). In relation to functionality as TRNG, on the other hand, the two LSBs of the counters are the ones that provide the most appropriate probability and entropy values.

The importance of these results compared to those obtained in the previous works is twofold since, in addition to considerably increasing their statistical significance, they demonstrate that different configurations of the same RO behave like different ROs, thus validating the strategy applied when designing the version 2.0 of the PUF/TRNG IP module proposed in this paper.

### 4.3. Output Bit Characterization

Once the bits that make up the output of the PUF/TRNG were determined, the test systems implemented in operation mode were subjected to different tests in order to evaluate a series of features directly related to the strength of the module (and, therefore, of the identifiers, keys, or random numbers generated with their help) against certain types of cyber attack. Basically, these tests aim to quantify the possible presence of bias in the outputs, analyze their randomness, and estimate the entropy they can provide.

**Figure 12:**
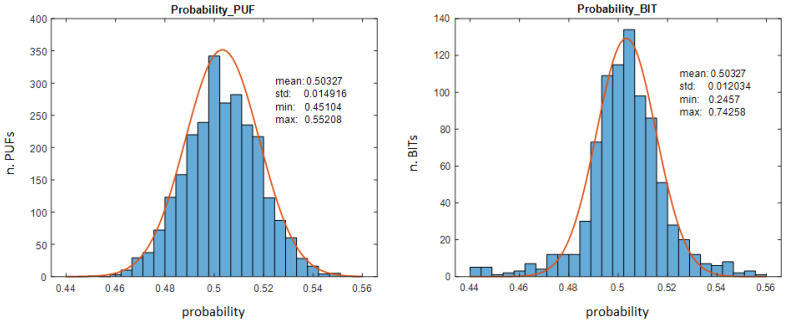
Probability of occurrence of 1 s in the output of each of the 2560 samples corresponding to the 256 configurations of the 10 IPs (**left**) and in each of the 960 output bits of all samples (**right**).

For PUF functionality, puf_statistics application allows us to statistically analyze the results of the different IP modules in a test system, based on the data obtained when invoking them (puf_getdata) or after an enrollment process (puf_enrollment), with the objective of determining the probability of occurrence of 0 s and 1 s at the outputs of the different samples (uniformity) and in the positions corresponding to the different bits (bit−aliasing). The histogram on the left of Figure 12 shows the probability that the output bits of each of the 10×256 samples take the value ‘1’, while the one on the right illustrates the probability that each of the 960 output bits (four for each RO comparison) takes the value ‘1’ for all 2560 samples.

It can be seen that the first histogram corresponds to a Gaussian distribution, with a mean very close to the ideal value of 0.5 and a small standard deviation, which ensures that the output of the PUFs implemented with this structure will not present a significant bias towards any of the two logical values, which could compromise the security of any cryptographic operation based on said outputs. On the other hand, the histogram that represents the probability of obtaining 1s in each of the output bits of the 2560 samples does not show such homogeneous results, since the data are distributed in a larger range of values, although most of the outputs show a Gaussian distribution with a mean and standard deviation similar to those of the previous case, which allows us to predict acceptable entropy values, as we verify below.

A similar analysis carried out using the reference outputs of the PUFs, obtained after an enrollment process, does not show significant differences. It is also possible to verify that the behaviors do not depend in a decisive way on the different run-time options of the PUF (LH, BG) or on the number of bits of the counters.

The availability of a high number of samples associated with the different configurations of the ROs allows us also to analyze the unpredictability of the module outputs, when they act as a PUF, using tools initially planned for the study of TRNGs. This is the case for the randomness tests provided by the NIST SP 800-22 standard [65] or the entropy estimation procedures defined in the NIST SP 800-90 recommendation [66].

The NIST test suite, available in [67], includes 15 statistical tests that can be applied to a binary sequence to compare it with a truly random sequence. In each test, the distribution of a relevant statistic under the assumption of randomness is calculated by mathematical methods. The hypothesis of randomness of the sequence being tested is accepted only when the statistical value of the data does not exceed a critical value of the same statistic for the reference distribution.

Throughout this work, NIST tests were used to analyze the randomness of the outputs of the IP module under development in three different situations: (1) instantaneous outputs of the PUFs with different run-time options; (2) reference outputs after an enrollment process with different run-time options; and (3) outputs of the module when it acts as a source of entropy with different options. The first two cases aim to evaluate the robustness of the PUF against possible attacks, while the third allows us to estimate the viability of the proposed structure to operate as a TRNG.

The length of the bit string generated by the puf4r5_1.0 IP (960 bits) limits the tests that can be applied in the first two cases to those that require the length of the bit string to be between 100 and 1000. In the third case, however, the number of calls to the module can be increased to concatenate the responses to obtain sequences of the size required by the other tests.

**Figure 13:**
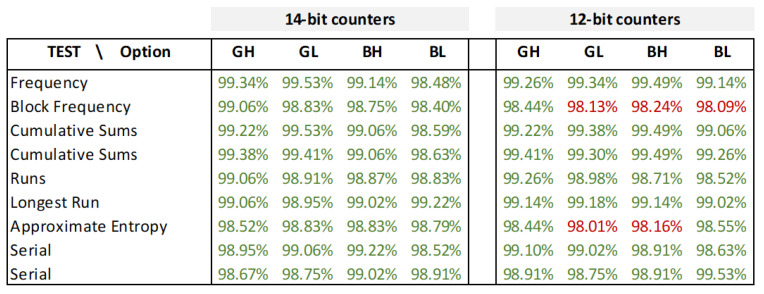
Percentage of sequences passing the subset of NIST SP 800-22 tests (values obtained from 2560 samples; green and red entries indicate, respectively, the cases in which the randomness hypothesis is accepted or not).

The data from the table in Figure 13 show what percentage of the 2560 sequences, corresponding to the instantaneous outputs of the 10 PUFs with 256 RO configurations implemented in test systems with 14- and 12-bit counters, passes the different tests for the four combinations of BG and LH run-time options. The results show that the randomness hypotheses raised in the different tests are accepted for all combinations of options when 14-bit counters are used, while some of them are rejected when 12-bit counters are implemented. The data for 10-bit counters (not included in the table) do reject the randomness hypotheses in most cases.

Similar results were obtained when the tests were applied to the same number of sequences and of the same size but obtained after performing an enrollment process on the different variants of the PUFs to calculate their reference outputs. Finally, 16 one million-bit strings, built by concatenating successive outputs from the different configurations and the different instances located in the test system, allowed us to verify that the IP module passes all the NIST randomness tests when acting as a TRNG.

In addition to describing how to design and test entropy sources, the NIST SP 800-90B recommendation provides a set of software applications that allow for determining whether or not an entropy source generates independent and identically distributed (IID) samples and estimate the min-entropy in each of these cases. Min-entropy is a measure of the lower bound of the unpredictability of PUF responses [68]. The results of applying the procedures provided by version 1.1.7 in [69] on the same input data used in the previous study (i.e., the 2.5 million-bit sequence formed by the concatenation of the 960 output bits of the 2560 PUF samples) are shown in Table 2. The input data do not pass the tests that would allow them to be considered IID, so the min-entropy (H_original) is estimated as the minimum value obtained from the ten estimation tests indicated in the first column of the table.

As can be seen, the minimum value is determined by the longest repeated substring (LRS) test for three of the four possible combinations of run-time options and by the compression test in the remaining case. Likewise, it can be verified that only the combinations using the Higher option to select the bits of the counters that are part of the PUF output reach entropy values per bit greater than 0.8. The results are very similar for implementations of the test system that use 12- and 10-bit counters in the comparison blocks, so they have not been included.

### 4.4. Performance Metrics Estimation

Uniqueness and reliability are the two main properties that define the quality of a PUF. While the first determines the ability of the PUF to uniquely identify the device on which it is implemented, the second indicates to what extent the output of the PUF is repeated in successive invocations. Both properties can be quantified by using as metrics the Hamming distances between outputs of a different (uniqueness) or the same (reliability) instance of PUF. The puf4r5_1.0 SDK includes two pairs of applications to calculate these metrics and estimate the compliance of both properties in the different PUF instances of a test system. The results obtained when the PUF behavior is evaluated by means of puf_HDinter and puf_HDintra applications considering the different run-time options are summarized in Table 3.

The data that appear in the second column of the table correspond to average HDinter values for the 10 PUFs and the 256 possible configurations of the ROs. For each configuration in each PUF, an enrollment process is performed in which the PUF is called 10 times to obtain its reference output. Subsequently, the Hamming distance between the reference output and 10 responses from each of the configurations of the other 9 PUFs is evaluated (a total of 1,024,000 calls to the PUFs). As can be seen, comparing each PUF with the other 2304 samples and obtaining the average values of all invocations makes HDinter practically independent from the chosen options and takes a value close to its ideal of 50%. To calculate the average, the values obtained after each PUF invocation have been normalized according to the expression $HDinter_{i}^{\prime }=ABS\left(50-HDinter_{i}\right)$ to avoid compensations of values above and below the ideal.

The average HDintra values for the 256 configurations and the 10 PUFs of the test system are shown in the third column of Table 2. Now, after performing an enrollment process similar to the one in the previous case to obtain the reference output of a PUF with a given configuration of the ROs, the Hamming distance against this reference output of 10 other responses of the same PUF with the same configuration is evaluated (204,800 calls in total). In this case, the results do depend on the options used when calling the PUF, so configurations that use Gray code counters and lower bits have smaller HDintra values. These results are consistent with the stability and entropy values obtained in Section 4.2 and also similar in terms of tendency to those described in [28] for a PUF with similar characteristics that does not exploit the configurability of ROs.

In addition to providing the reference output for a given PUF, the enrollment process carried out by puf_enrollment application allows for discarding a series of comparisons (challenges) between those that most negatively affect the reliability of the PUF. The discarded challenges are registered in a challenge selection mask, which can be used in subsequent calls to the PUF to eliminate these comparisons. Columns 4 and 5 of Table 2 reveal the usefulness of the challenge selection mechanism by illustrating, respectively, the HDintra values and the reduction percentages obtained by eliminating 10% of the comparisons in the enrollment process.

**Figure 14:**
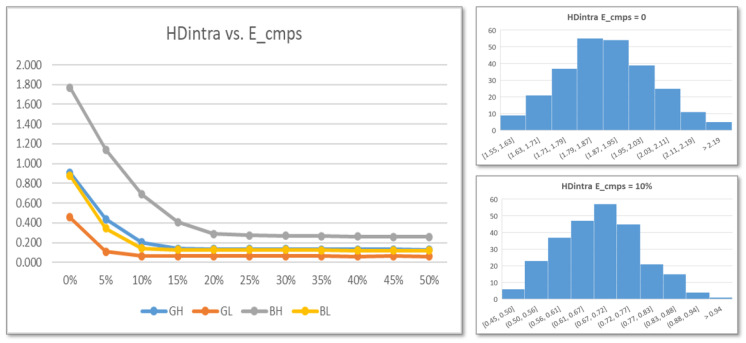
HDintra versus percentage of eliminated comparisons (**left**) and distribution of the average values of HDintra for the 256 configurations of ROs before and after applying the challenge selection mechanism (**right**).

The graph on the left of Figure 14 shows the evolution of HDintra values as a function of the percentage of eliminated comparisons for the four possible combinations of run-time options. As can be seen, the minimum value of HDintra is reached after eliminating 15% (GH, GL and BL) or 20% (BH) of the comparisons. On the other hand, the histograms on the right of Figure 14 show the distribution of the average values of HDintra corresponding to each of the 256 RO configurations before and after applying the challenge selection mechanism to discard the 10% of comparisons.

The tests performed to obtain the data shown in Table 3 and Figure 14 were run under standard operating conditions of voltage and temperature (1 V and 30 °C). It is well known that PUF metrics can be affected by variations in operating conditions that arise naturally or are forced by an attacker. The results previously obtained by the authors in an extensive study carried out on a PUF with a similar structure implemented on the same family of programmable devices show that the increase between the maximum and minimum values of HDintra when the temperature and voltage are varied within the operating range that appears in the FPGA manufacturer’s documentation corresponds to 0.31 and 0.42, respectively [28]. In the proposed PUF, the possible increase can be more than compensated for by the reduction obtained by applying the challenge selection mechanism.

HDintra and HDinter provide statistical measures of the repeatability and variability of the PUF responses, which are directly related to their reliability and uniqueness properties, respectively. However, to more realistically estimate the usefulness of the PUF, when combined with a helper data algorithm (HDA), as a basic element for the generation and recovery of secret keys, puf_reliability and puf_uniqueness applications available in the puf4r5_1.0 SDK can be used.

To evaluate the reliability of the PUFs included in the test system, puf_reliability first performs an enrollment process for each PUF and each configuration to obtain its reference output and, subsequently, analyzes the key masks obtained by applying an ECC, with a given repetition factor, to the responses of the successive series of invocations to the PUF. Similarly, to estimate the uniqueness of the PUFs included in the test system, puf_uniqueness also performs an enrollment process for each PUF and configuration to obtain its reference output, to subsequently analyze the differences between the key mask obtained when applying an ECC with a repetition factor given to this reference output and those corresponding to the responses of the successive series of invocations to the other configurations and PUFs of the test system.

The tests carried out to determine the reliability and uniqueness of the 256 configurations of each of the 10 instances of the test system revealed that by discarding 10% of the challenges and using a repetition factor of 9, it is possible to recover a 96-bit key for all combinations of run-time options. With the number of bits supplied by the PUF, it is only possible to process 128-bit keys with the GH and GL options. On the other hand, keys obtained with a given PUF and configuration can never be recovered with another configuration of the same PUF or by a different PUF, even if the ECC repetition factor is increased.

In addition to providing valuable information on the behavior of the basic components of the PUF/TRNG module, the results obtained from the experiments carried out on the test systems that incorporate the puf4r5_1.0 IP show that different configurations of the proposed CRO present properties similar to those of ROs used in previous versions of the IP module. This led us to confirm the hypothesis raised when addressing the design of the puf4r5_2.0 IP in relation to the combined use of different configurations to reduce resources and increase the size of the module output.

## 5. Test System for Performance Evaluation of the Proposed PUF/TRNG

**Figure 15:**
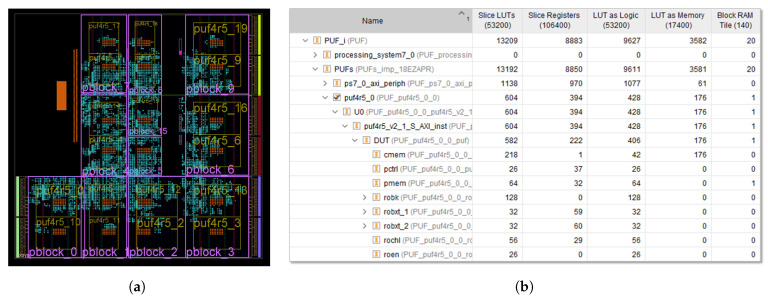
Test system to evaluate the performance of the PUF: (**a**) Distribution of 20 instances of the PUF on the programmable device. (**b**) Resource consumption of the test system and the different components of the puf4r5_2.0 IP module.

To characterize the behavior of the new PUF/TRNG proposal and evaluate its performance, an additional series of test systems similar to that shown in Figure 15 were implemented using different design options. These test systems include puf4r5_2.0 IP modules in characterization and operation mode that use 10, 12, and 14 bits in the counters involved in the comparison between the RO frequencies. The functions and applications of the SDK were also adapted to the structure of the new IP module to facilitate the validation and performance estimation stages using the processing system included in the ZYNQ XC7Z020 device of the Pynq-Z2 board [61].

An array of only 4×4 CLBs is now sufficient to host 32 CROs (equivalent to 32×256=8192 ROs) capable of providing up to 32K bits at the output of the PUF/TRNG when implemented in operation mode. Reducing the number of CLBs required by the RO bank by a factor of 7.5 allows us, in this case, to include 20 instances of the IP in each test system. As in the test system described in Section 4, the locations of the CRO banks are defined when instantiating the different copies of the IP, while ‘pblock’ directives are used to guide the synthesis tool when implementing the remaining components of each IP. With the parameters used, each of the IP instances requires one Xilinx Series 7 Block RAM (BRAM) to implement the module output memory in operation mode and eight BRAMs in characterization mode. This type of primitive limits to 17 the maximum number of instances that can be included in the device available on the Pynq-Z2 board when the test system is implemented in characterization mode.

### 5.1. PUF/TRNG_2.0 Bit Selection

**Figure 16:**
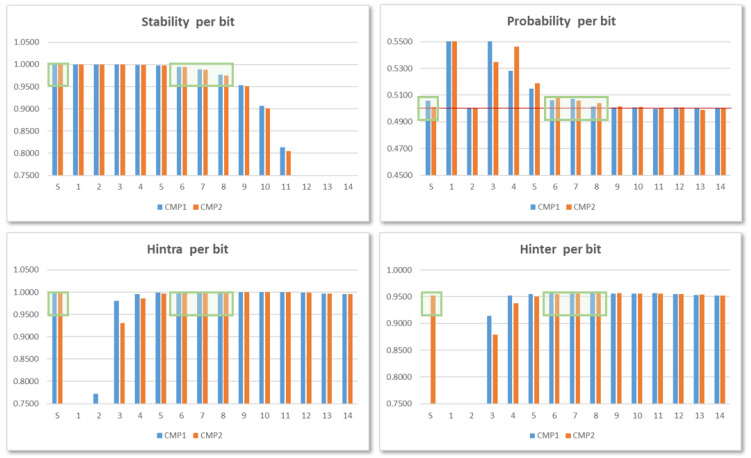
Stability, probability, and entropy metrics calculated for each bit of the counters (average values for one hundred calls to all PUF/TRNG instances and RO configuration options, with the two types of counters; green boxes point out the most suitable bits to construct the output for PUF functionality).

To verify that the bit selection criterion applied in the version of the PUF/TRNG that considers each configuration independently is still valid when several configurations are combined within the same module, a procedure similar to that described in Section 4.2 has been followed to analyze the output bits resulting from successive comparisons between ROs in the different IP instances included in test systems implemented in characterization mode with 10-, 12-, and 14-bit counters. Figure 16 shows the stability, probability, and entropy values associated with each bit when 14-bit counters are used in the comparisons. The results correspond to the average of the data obtained by using puf_bitselect application to invoke 10 times each of the 17 puf4r5_2.0 IPs in the test system, with the maximum number of comparisons (8192) and using binary and Gray code counters.

When comparing these graphs with those shown in Figure 11, it can be seen that there is a great similarity in the behavior of the different metrics, although the number of samples and the length of the output differ considerably in both cases. The sign bit provides good values in all the metrics only for comparisons between ROs implemented in LUTs located in different CLB positions (COMP2), while bits 6-8 present acceptable values for both comparisons, thus corroborating the choice made in previous versions of the PUF/TRNG module.

### 5.2. PUF Performance Metrics

**Figure 17:**
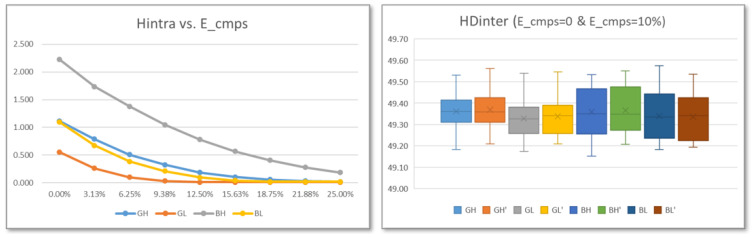
Evolution of HDintra versus percentage of eliminated challenges (**left**) and distribution of HDinter before and after (’) removing 10% of the challenges (**right**) for the different combinations of run-time options.

The graphs on the left of Figure 17 show the evolution of the average HDintra values for the 20 PUFs of the test system with 14-bit counters implemented in operation mode when using the different Binary/Gray (BG) and Lower/Higher (LH) options and a progressive number of challenges is eliminated in the enrollment process. For each combination of options, each PUF is called 100 times during enrollment and 100 times more to calculate the Hamming distance from the reference output. It can be verified that even using outputs of the maximum length provided by the PUFs included in the test system, that is, 32K bits, the value of HDintra is less than 0.1 after eliminating 6% (GL), 15% (GH and BL), or 25% (BH) of the comparisons. The values obtained are very similar regardless of the number of bits of the counters, although, in general, HDintra increases slightly when the size is reduced.

To analyze whether the challenge selection mechanism can negatively affect the uniqueness of the different PUFs, a set of tests was carried out aimed at calculating the average HDinter values for the 20 PUFs of the test system when the Binary/Gray (BG) and Lower/Higher (LH) options are used. For each combination of options, an enrollment process is carried out in which the PUF is called 100 times to obtain the challenge selection mask and the reference output, discarding 10% of the comparisons. Subsequently, using the obtained selection mask, the Hamming distance between the reference output and 10 responses of each of the other 19 PUFs is evaluated.

The box-and-whisker diagram on the right of Figure 17 shows the distribution of HDinter values for different run-time options, before and after applying the challenge selection mechanism to discard 10% of comparisons. It can be seen that the average values of HDinter are in this case greater than 49.3, which means that practically half of the bits change from one PUF to another of those instantiated in the test system. Furthermore, HDintra values vary only slightly (0.1–0.2% for different options) depending on whether or not the challenge selection process is carried out to improve the reliability of the system.

As an alternative to using puf_reliability and puf_uniqueness to estimate the performance of the PUFs included in the new test system, we use the puf_keygen application, provided in the puf4r5_2.0 SDK, to demonstrate the ability of the PUF/TRNG modules to obfuscate and recover cryptographic keys when they are part of the secure key management system described in Section 6.

### 5.3. PUF Response Time

**Figure 18:**
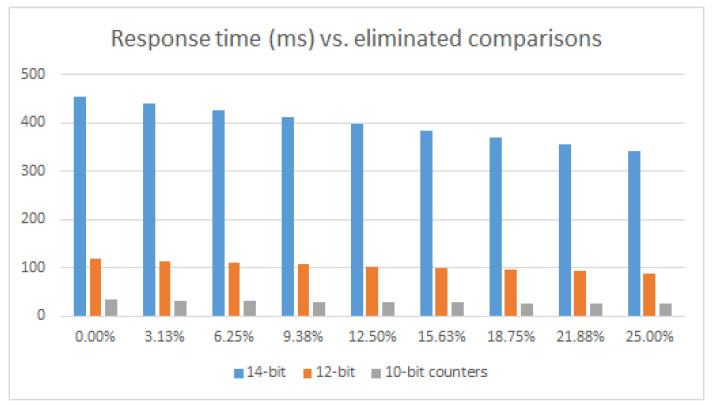
Response time (in ms) versus percentage of comparisons eliminated by the challenge selection mechanisms for 8192-RO PUFs included in test systems with 10-, 12-, and 14-bit counters.

The time taken by the PUF to provide the response depends primarily on the characteristics of the programmable device on which it is implemented (which determine the average oscillation frequency of the ROs), the parameters used when implementing the IP module (which define the maximum number of comparisons and the number of clock cycles per comparison), and the run-time options used when invoking it (which select the effective number of comparisons). For illustrative purposes, Figure 18 shows the variations in response times for one of the PUFs included in the three test systems implemented in operation mode on the Pynq-Z2 board versus the percentage of comparisons eliminated by the challenge selection mechanism. As can be seen from the study carried out in Section 4.1, the average oscillation frequency of the ROs used is about 355 MHz. When 14-bit counters are used, the PUF requires a minimum of 380 ms to complete the 8192 comparisons. The values shown in the graph also include the time that the processing system spent sending the challenge selection mask and accessing the PUF output through the AXI4-Lite bus. These tasks represent an increase in response time of just over 15% when 14-bit counters are used and 25% for 10-bit counters. The graphs also show that response times are reduced by just under half every time the counter size is reduced by one bit.

### 5.4. TRNG Validation

The trng_getdata application provides a systematic mechanism to obtain the data necessary to analyze the correct operation of the proposed IP module as a true random number generator and estimate its entropy, using the tools provided by NIST, as a function of the different design parameters and run-time options. In combination with trng_validation, it also allows ‘Health Tests’ to be applied to ensure that the entropy source continues to have the appropriate properties at a later point in the lifetime of the circuit. The tests carried out on the test systems that incorporate the proposed IP are intended to (1) generate the input files for the tests and applications provided by NIST to analyze the randomness and estimate the entropy; (2) execute the tests proposed in NIST-SP800_22R1a; (3) run the entropy evaluation applications available in SP800-90B_EA-1.1.7; and (4) once the appropriate parameters have been calculated, run the health tests proposed by NIST.

To perform the randomness analysis, the minimum number of bits required by the procedures provided by NIST to evaluate the entropy associated with the source (one million bits) was captured for each of the 20 IPs in the test systems. Although NIST tests allow successive TRNG outputs to be concatenated to form longer sequences, the original structure of the data was maintained as 31 sequences of 32,768 bits. The size of the sequences is not sufficient to run some of the randomness tests. However, the tests carried out provide important information about the behavior of the module depending on the counter size and the used options. The results obtained show that when 14-bit counters are implemented, all PUFs mostly pass the 11 tests whether the binary or Gray code outputs are considered (only three of the 440 cases analyzed fail). The results are similar for 12 bits, except in the case of the FFT test for Gray code counters.

Using the same data as in the previous analysis and the procedures provided in the recommendations included in NIST SP 800-90, it is possible to estimate the entropy of the different TRNGs and determine whether they present IID or non-IID behavior. The results for 14-bit counters show that the TRNGs behave as an IID source of entropy in all cases except one. However, the estimated average entropy values (0.99553 and 0.99478, for Gray and binary counters, respectively) do not allow for passing the health tests proposed in the NIST SP 800-90 recommendation. The situation changes if the entropy is estimated considering sources with non-IID behavior (0.86078 and 0.85106). In this case, if the mean value of the entropy of the 20 TRNGs for each type of counter is set as the target entropy, the health tests are passed in 39 of the 40 cases analyzed.

The entropy of the three options considered when 12-bit counters are used (Gray, Gray + XOR post-processing, and binary) was estimated taking into account non-IID behavior. When using Gray code counters, it is necessary, in this case, to use twice as many TRNG calls and apply the XOR operation to achieve entropy values greater than 0.8. The health tests fail in seven cases when the average values of the entropy for each type of counter are defined as objective and only in one of the 40 cases when the minimum values are taken. Finally, using 10-bit counters, it is only possible to achieve, in the best of cases, an entropy per bit of the order of 0.3, which makes this alternative unfeasible in applications that intend to use the TRNG functionality of the proposed module.

### 5.5. Summary and Result Comparison

Combining the results obtained when evaluating the performance of the IP module when it acts as a PUF with the statistical analysis of its basic structure carried out in Section 4, it is possible to obtain some interesting conclusions from the point of view of the practical use of the proposed PUF/TRNG IP:

- The uniqueness of the PUF is excellent regardless of the design parameters and run-time options used, as demonstrated by the fact that the average HDinter values are greater than 49.3 for 12- and 14-bit counter implementations, as well as the circumstance that they remain almost unchanged when the challenge selection mechanism is applied to improve PUF reliability.
- The reliability of the PUF does depend on the run-time options that select the type of counter (binary or Gray code) and the bits chosen to be part of the output (lower or higher). The combinations GL and BH always show the most favorable and unfavorable values of HDintra, respectively, while GH and BL provide intermediate values of this metric.
- Regardless of the used options, the uniformity of the PUF outputs (both of the test system for characterization analyzed in Section 4 and of the test system that uses the proposed PUF) reveals the absence of bias that could compromise the security of a system that uses the latter as a basic primitive. However, the tests carried out to characterize the different combinations of run-time options in the test systems revealed that those combinations that use lower bits of the counters do not provide the necessary entropy to ensure the strength of the PUF against certain types of attacks.
- Depending on the application or task in which it will be used, the user can prioritize the reliability or strength of the PUF. Without forgetting that reliability can also be improved by properly applying the challenge selection mechanism provided by the puf4r5_2.0 SDK, the use of GH options can constitute an appropriate trade-off.

**Figure 19:**
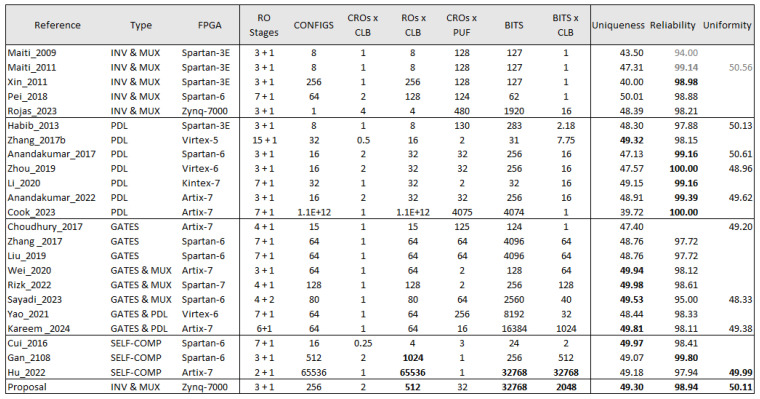
Comparative table of different configurable RO-PUFs proposed in the literature. Hardware efficiency is given by the number of ROs per CLB and the number of output bits per CLB. The values of uniqueness, reliability and uniformity allow the performance of each proposal to be contrasted. (Maiti_2009 [32], Maiti_2011 [33], Xin_2011 [34], Pei_2018 [36], Rojas_2023 [28], Habib_2013 [37], Zhang_2017b [38], Anandakumar_2017 [39], Zhou_2019 [40], Li_2020 [41], Anandakumar_2022 [42], Cook_2023 [43], Choudhury_2017 [45], Zhang_2017 [46], Liu_2019 [47], Wei_2020 [49], Rizk_2022 [50], Sayadi_2023 [52], Yao_2021 [53], Kareem_2024 [54], Cui_2016 [56], Gan_2018 [57], Hu_2022 [58]).

The table in Figure 19 allows us to compare our proposal with other configurable RO PUFs published in recent years. To facilitate the analysis, the different proposals have been grouped into the four basic types described in Section 2, although some of them combine ideas or characteristics from more than one of the groups. In the left part of the table, information appears on the type of configurable RO-PUF and the family of programmable device corresponding to each of the referenced proposals.

The central part of the table gathers data on the size of each PUF (number of CRO stages, number of CROs, and output bits), as well as some indicators of the hardware efficiency of its implementation (ROs/CROs per CLB, and bits per CLB).

PUF performance metrics are shown in the right part of the table. Uniqueness and uniformity are expressed, respectively, by the averages of the Hamming distances between the outputs of different PUF instances (HDinter) and the average of the Hamming weights of these outputs. To unify the notation with respect to that used by other authors, reliability has been calculated as the difference between the ideal value, 100%, and the average HDintra in successive invocations of the same PUF.

As can be seen, our proposal presents a hardware efficiency only surpassed by the structure analyzed in [58], while it provides values of the three performance metrics comparable to those of best proposals in the state of the art, without any of them managing to surpass it in the set of the three metrics.

## 6. Using the PUF/TRNG IP for Secure Key Management

One of the characteristics that has made PUFs a fundamental element to increase the security of resource-limited embedded electronic systems is their usefulness for managing secret information of an ephemeral or permanent nature, linking it uniquely to the device that implements the PUF and without the need of storing it in expensive nonvolatile memories. The fact that the reliability of a silicon PUF is not absolutely guaranteed even with the use of challenge selection techniques that allow it to increase considerably requires the use of HDAs [70] that incorporate different types of ECCs [71].

**Figure 20:**
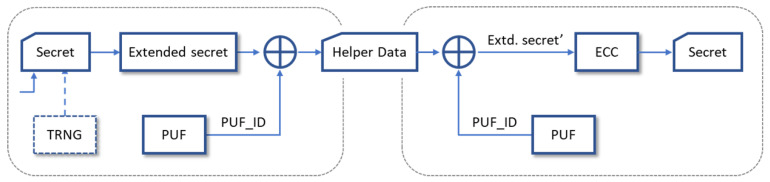
Secret obfuscation/recovery scheme using the proposed PUF/TRNG module and a repetition-based ECC.

Figure 20 shows a secret obfuscation and recovery scheme using a simple repetition-based ECC. The secret, supplied by the user, provided by a key generation algorithm, or obtained randomly using the TRNG functionality of the IP module, is first extended, replicating RC times each of its bits, to be then XOR-ed with an equivalent number of bits taken from the PUF output. The helper data obtained as a result of this operation do not reveal information about the secret and do not allow it to be recovered on another device because they are linked to the device that generated them. For these reasons, their contents are not sensitive from a security point of view and therefore do not require special storage conditions.

Helper data are, however, essential to recover the secret on the same device on which it was obfuscated. To implement this, first, an XOR operation is performed between the helper data bits and an equivalent number of bits from a new PUF invocation. The output of the PUF may vary slightly from that used when obfuscating the secret, but the use of an ECC with a repetition factor equal to that used in the obfuscation phase allows these errors to be corrected and the secret properly recovered as many times as necessary.

In order to analyze the capacity of the proposed module to generate and recover secret keys of different lengths, an extensive set of tests was carried out with the help of the puf_sysgen application. This application receives as input the length of the secret (KEY), the repetition factor (RC) of the ECC, and the number of comparisons that are performed in the PUF (CMPs). After verifying that it is possible to carry out the obfuscation process with the indicated parameters, the application calculates the maximum number of comparisons that can be eliminated in a subsequent enrollment process in which the reference output and the corresponding challenge selection mask are generated.

Following the scheme in Figure 20, this reference output is used, together with an extended version of the secret, to calculate the helper data that allow the secret to be recovered later. puf_keygen also allows for the estimation of the reliability of the PUF for secret key management by facilitating the iterative call to the PUF to recover secret information using the challenge selection mask and the helper data obtained when obfuscating it.

**Figure 21:**
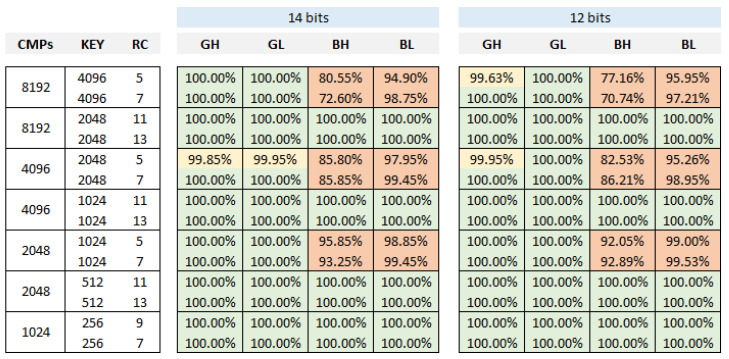
Reliability in recovering secrets of different lengths, varying the number of comparisons and the ECC repetition factor, for the four combinations of run-time options and 12- and 14-bit counters (data show normalized values from a total of 2000 cases corresponding to 100 recoveries in each of the 20 PUFs in the corresponding test system).

The most significant results of the tests carried out are summarized in the table that appears in Figure 21. A total of 14 scenarios were considered, using between 25% and 100% of the available comparisons to obfuscate and recover keys of different sizes (256 to 4098 bits) using repetition factors of 5 to 13. The first three columns on the left show, respectively, the number of comparisons, the length of the key, and the ECC repetition factor used in the obfuscation and recovery processes of secret information. The remaining columns show the percentage of occasions in which the secret is successfully recovered for each combination of the run-time options of the 20 PUFs in the test systems with 14- and 12-bit counters. For each PUF and each combination of options, 10 invocations were made in the enrollment process and 100 recovery attempts were carried out (i.e., 2000 attempts per test system). The green color means that no errors have occurred in any of the recoveries of any of the PUFs, while yellow and red represent, respectively, cases in which a small or a high number of recovery errors have occurred in some PUF of the test system.

The results show that it is possible to recover keys of up to 2048 bits in the 20 PUFs of the test systems with all combinations of run-time options and up to 4098 bits with the options using Gray code counters (GH and GL).

## 7. Conclusions

Taking full advantage of the CLB components of Xilinx Series 7 programmable devices to implement configurable ring oscillators, the PUF/TRNG structure proposed in this work allows for considerably increasing the efficiency of the hardware design, multiplying by 128 the number of ROs offered by the preceding versions of similar PUFs previously proposed by the authors. This increase in hardware efficiency has been used in this work to pursue a double objective: on the one hand, to have a number of samples that allows us to perform a rigorous and significant study on the statistical properties of the results of the proposed PUF/TRNG structure; on the other hand, to design a new IP module that allows for simultaneously increasing the size of the output and reducing the amount of resources necessary for its implementation in programmable devices.

To meet both objectives, the work describes the development of two IP modules that incorporate the proposed configurable RO blocks. Both IPs share an RO comparison and bit selection strategy that allows four bits to be obtained for each of the available ROs, incorporate a standard interface to connect to a general-purpose processor, and implement a challenge selection mechanism that allows for reducing significantly the variability of successive responses. The main difference between both IPs is that while in the first one, the configuration of the ROs is defined through the external interface, with the idea of analyzing the behavior of the different configurations, the second one internally combines different configurations as part of the challenge sequence generation process to increase the size of the module response.

Two series of test systems, instantiating several copies of each of the two IPs, have been implemented using different design parameters (operating mode and size of the counters used to compare the oscillation frequencies). In addition, the corresponding SDKs have been adapted and completed with the aim of speeding up the execution of the planned experiments to carry out the statistical characterization and performance evaluation of the prototypes on the development boards themselves.

The test systems for the first IP include, in both modes of operation, 10 instances of the PUF/TRNG with a bank of CROs that occupies 8×15 CLBs, allowing the analysis of a total of 2560 samples with 960-bit outputs. The availability of a high number of samples allowed a significant statistical study to be carried out on the properties of the proposed structure, both when the PUF (uniqueness, reliability, and unpredictability) and TRNG (randomness and entropy) functionalities are used.

In addition to facilitating the analysis of the oscillation frequencies of the proposed configurable ROs and corroborating the bit selection criterion proposed by the authors in previous works, the results obtained allowed us to confirm that the PUFs and TRNGs that include the proposed structure present, for each possible configuration of the CROs, a behavior very similar to that of the previous versions of the IP module. This reinforces the idea, applied in the design of the new IP module, that it is possible to combine different CRO configurations to build a PUF/TRNG that requires fewer resources and provides more output bits.

The test systems built to evaluate the performance of the proposed PUF/TRNG module incorporate 17 (characterization mode) or 20 (operation mode) IP instances that use a bank of CROs that occupies only 4×4 CLBs to provide 32K output bits. The number of samples available does not allow us, in this case, to obtain results on the randomness or entropy of their outputs when the IPs are used as PUFs. However, the results are very different when IPs act as entropy sources. In this case, whether the data from the 20 TRNGs are combined (concatenating the outputs to obtain sequences of one million bits) or if the TRNGs are analyzed individually, both the NIST randomness and the entropy health tests are largely passed.

For PUF functionality, the histograms that represent the probabilities of obtaining 1s in the 32K bits of the outputs of the 20 PUFs of the test systems show Gaussian distributions with means very close to 0.5, which suggests the absence of biases that could reduce the robustness of the PUF, compromising unpredictability and making it vulnerable to certain types of cyber attacks. The uniqueness of the responses of different PUFs is guaranteed, since the average values of HDinter remain above 49.3 for the different combinations of run-time options. Furthermore, these values vary only slightly (±0.1%) after the enrollment process. Finally, the considerable increase in the output size between the two considered IPs (960 versus 32,786 bits) causes the HDintra values to slightly increase in the proposed module. However, the reliability of PUFs can be improved by using the challenge selection mechanism described in the work. For the case implemented in test systems, it is possible to obtain HDintra values below 0.1 by eliminating between 6% (GL) and 25% (BH) of the possible comparisons.

In addition to providing a practical and complete solution for the incorporation of security elements in SoCs implemented on programmable devices, when compared with other state-of-the-art proposals published in recent years, the PUF/TRNG IP module described in this work outperforms most of them in hardware efficiency, also providing values for the three main performance metrics similar to those of the best proposals, without any of them outperforming it in the set of the three metrics. Consequently, as illustrated by the study of its ability to generate and recover hardware-linked cryptographic keys, the inclusion of this IP module in a key management system allows handling 256-, 512-, 1024-, and 2048-bit keys with all combinations of run-time options, and 4096-bit keys with GH and GL options.

## Figures and Tables

**Table 1:** Main functions and applications included in the SDK for puf4R5 IP modules.

Function	Description
PUF_createMMIOWindow	Create memory-mapped IO window for PUF/TRNG registers
PUF_enrollment	Generate PUF reference output and challenge selection mask
PUF_writeChallegesMask	Write challenge selection mask
PUF_applyChallenges	Reset, configure and start PUF/TRNG operation
PUF_readOutput	Read PUF/TRNG results from output memory
**Application**	**Description**
puf_bitselect	Select counter bits that form the PUF output (characterization mode)
puf_getdata	Capture data for off-line evaluation of PUF performance (operation mode)
puf_statistics	Calculate parameters to estimate the unpredictability of PUF outputs
puf_HDintra, puf_HDinter	Obtain metrics related to PUF reliability and uniqueness
puf_reliability, puf_uniqueness	Evaluate reliability and uniqueness of the PUF when used for ID generation
puf_test	Combine some of the above functions to illustrate PUF operation
puf_keygen *	Demonstrate PUF ability to obfuscate and recover cryptographic keys
trng_getdata	Collect data for randomness analysis and entropy estimation as TRNG
trng_validation	Run health test of the entropy source provided by the IP acting as TRNG

* Only in puf4r5_2.0 SDK.

**Table 2:** Use of NIST SP 800-90B tools to estimate the entropy associated with the PUFs of the test system implemented with 14-bit counters for the different combinations of run-time options. Min-entropy is estimated as the minimum value (in bold) obtained from the different estimation tests.

Test/Options	GH	GL	BH	BL
Most Common Value	0.99527	0.99461	0.98815	0.97661
Collision Test	0.92264	0.92292	0.91741	0.93008
Markov Test	0.99722	0.99622	0.98987	0.98123
Compression Test	0.87331	0.85969	**0.83169**	0.85284
T-Tuple Test	0.93558	0.93134	0.93134	0.91799
LRS Test	**0.86169**	**0.40777**	0.98442	**0.62947**
MultiMCW Prediction Test	0.99150	0.99305	0.99511	0.98333
Lag Prediction Test	0.97968	0.98100	0.98050	0.98005
MultiMMC Prediction Test	0.99489	0.99113	0.98890	0.97663
LZ78Y Prediction Test	0.99572	0.99497	0.98830	0.97662
**H_original**	**0.86169**	**0.40777**	**0.83169**	**0.62947**

**Table 3:** Average values of quality metrics for the PUFs in puf4r5_1.0 test system.

Run-Time Option (BG-LH)	HDinter (Mean)	HDintra (All cmps)	HDintra (−10 % cmps)	Reduction (%)
Gray/Higher	48.62	0.95	0.20	79.12
Gray/Lower	48.62	0.48	0.07	86.27
Binary/Higher	48.64	1.88	0.69	63.30
Binary/Lower	48.61	0.93	0.14	84.63

## Data Availability

The test systems and applications used to validate and evaluate the performance on a Pynq-Z2 development board of the CRO-PUF/TRNG IP module proposed in this paper are openly available on GitLab at [https://gitlab.com/hwsec/cro_puf-trng_r5] (accessed on 19 July 2024), so that the reader can perform different experiments on his own development board to analyze the effect of different design parameters on the ability of the PUF/TRNG module to act as an essential element for obfuscation and retrieval of secret information in a key management system.

## Abbreviations

The following abbreviations are used in this manuscript:

AXI	Advanced extensible interface
BR PUF	Bistable ring physical unclonable function
BRAM	Block random-access memory
CLB	Configurable logic block
CRO	Configurable ring oscillator
DRAM	Dynamic random-access memory
ECC	Error-correcting code
FPGA	Field-programmable gate array
GUI	Graphical user interface
HDA	Helper data algorithm
IC	Integrated circuit
ID	Identifier
IID	Independent and identically distributed
IoT	Internet of Things
IP	Intellectual property
LFSR	Linear feedback shift register
LSB	Least significant bit
LSR	Longest repeated substring
LUT	Look-up table
MSB	Most significant bit
NIST	National Institute of Standards and Technology
PDL	Programmable delay line
PL	Programmable logic
PS	Processor system
PUF	Physical unclonable function
PYNQ	Python Productivity for Zynq
RO	Ring oscillator
RoT	Root of trust
SoC	System on chip
SDK	Software development kit
SRAM	Static random-access memory
TMV	Temporal majority voting
TRNG	True random number generators
